# Daily sitting time and hyperuricemia in US adults: A dose-response association with the mediation effect of renal function-related indicators (NHANES 2007–2018)

**DOI:** 10.1097/MD.0000000000046026

**Published:** 2026-01-30

**Authors:** Li Li, Fei Wang, Qifang Guo

**Affiliations:** a Department of Medicine, People’s Hospital of Ningdu County, Ganzhou, Jiangxi, China; b Department of Rheumatology and Immunology, The Second Affiliated Hospital, Jiangxi Medical College, Nanchang University, Nanchang, Jiangxi, China.

**Keywords:** daily sitting time, function-related indicators, hyperuricemia, mediation analyses, physical activity

## Abstract

The rising prevalence of hyperuricemia (HUA) and prolonged sitting time (ST) has been recognized as a significant public health concern globally. However, the extent to which ST is associated with HUA via renal function-related indicators remains unclear. The study included 25,470 participants aged 20 and up from the National Health and Nutrition Examination Survey 2007 to 2018. Based on ST, participants were separated into 4 groups: <4 hours group, 4 to 6 hours group, 6 to 8 hours group, and ≥8 hours group. Logistic regression and subgroup analysis were utilized to examine the association. Restricted cubic spline analysis was employed to investigate the nonlinear correlations. Finally, to ensure the stability and reliability of the results, interaction, trend test and mediation analysis were further conducted. The logistic hierarchical regression analysis revealed a higher probability of HUA in the 4 to 6 hours sedentary time group for physical activity (PA) <400 metabolic equivalent (MET)-min/week (all *P* < .05). However, for PA ≥ 400 MET-min/week, there was no significant difference (all *P* > .05). There was an inverted U-shaped relationship between daily ST and HUA (*P* nonlinear <.05). The results of the mediation analysis showed that blood urea nitrogen mediated 17.06% of the relationship between daily sitting duration and HUA, serum creatinine mediated 27.18%, and estimated glomerular filtration rate mediated 32.50%. All these factors showed insufficient mediating effects (all *P* mediation <.05). The study demonstrated that the prevalence of HUA increases with longer daily ST, particularly in individuals who engage in <400 MET-min/week of PA. There was an inverted U-shaped relationship between sitting duration and HUA. Serum creatinine, blood urea nitrogen, and estimated glomerular filtration rate were significant mediators in this relationship.

## 1. Introduction

Hyperuricemia (HUA) is a widespread condition around the world. The prevalence of HUA has been rising globally, with a declining age of onset in recent years.^[1]^ Between 2007 and 2016, the United States experienced a high incidence of approximately 20%.^[2]^ HUA is primarily a metabolic condition mostly caused by a purine metabolism issue, which results in an excessive increase in serum uric acid (SUA) levels in the body. Uric acid can have an impact on the body’s health since it acts as both an antioxidant and a prooxidant.^[3]^ HUA, characterized by elevated uric acid levels, is considered a primary precursor to gout, an inflammatory ailment.^[4]^ Additionally, HUA is linked to a variety of chronic metabolic diseases, including hypertension,^[5]^ diabetes,^[5]^ and chronic kidney disease (CKD).^[6]^ A longitudinal cohort study found that higher baseline SUA levels significantly elevated the risk of metabolic syndrome, with a dose-response relationship seen in both men and women.^[7]^ Furthermore, research has revealed that elevated SUA levels are an independent risk factor for cardiovascular morbidity and mortality,^[3]^ as well as worse outcomes in individuals with heart failure.^[8]^

Sedentary behavior (SB) for long periods of time has serious health hazards, and this trend has become more common with lifestyle changes over the past century. According to the Sedentary Behavior Research Network, SB is defined as any awake behavior involving an energy expenditure of 1.5 metabolic equivalent (METs) or less while sitting, reclining, or lying down.^[9]^ SB has been reported to increase adult all-cause and cardiovascular disease mortality rates and to be a risk factor for a variety of diseases, including diabetes, dementia, cancer, and cardiovascular disease.^[10–14]^ Additionally, SB negatively impacts mental health and overall quality of life. Studies have established a link between prolonged sedentary time and an increased risk of anxiety and depression.^[15]^ In addition, sedentary habits can contribute to decreased health-related quality of life, impairing mobility, self-care, and daily activities. Therefore, the rising prevalence of HUA and prolonged sitting time (ST) should be considered serious public health issues.

Studies have demonstrated that SB impairs muscle contraction and blood flow, leading to endothelial dysfunction and increased vascular resistance, which adversely affects kidney function.^[16,17]^ Furthermore, renal failure leads to the elevation of SUA levels because the kidneys are the primary organs responsible for the excretion of SUA. Notably, despite these intriguing potential, there is a clear lack of research into this trade-off role. Additionally, there is a shortage of large sample data analysis related to this issue in the US. Therefore, this study investigated the association between daily ST and HUA, performed interaction analysis of physical activity (PA), and analyzed renal function indicators as mediators for the 1st time.

## 2. Methods

### 2.1. Study population

**Figure 1.**
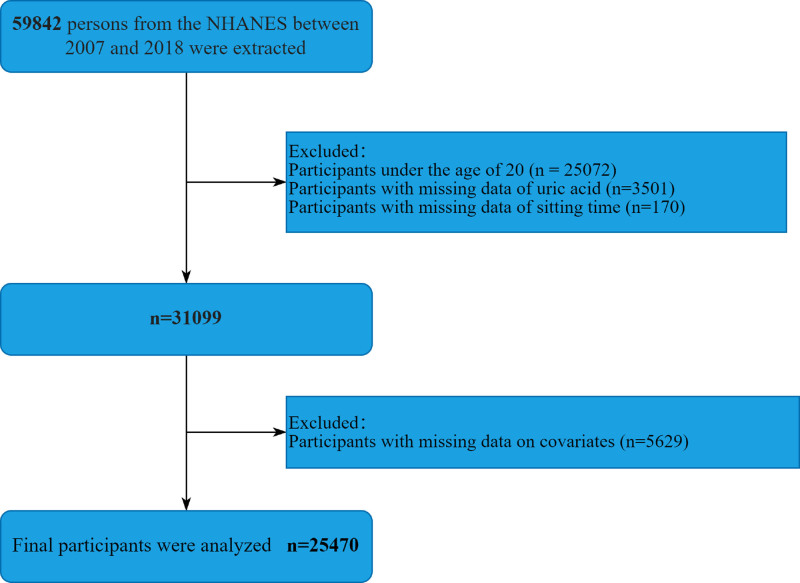
The detailed screening procedure for this study. NHANES = National Health and Nutrition Examination Survey.

Six cycles and a total of 59,842 individuals’ data of the study were collected from the National Health and Nutrition Examination Survey (NHANES) 2007 to 2018, a continuous, countrywide, biennial, cross-sectional survey that collects demographic, socioeconomic, nutritional, and health-related data from the US population. The National Center for Health Statistics Ethics Review Committee authorized the research strategy, and all participants provided informed consent through documentation. The study adhered to the 1975 Declaration of Helsinki. This study eliminated participants under the age of 20 and those with missing data on uric acid, ST, or other variables. Figure 1 summarizes the detailed process for selecting participants.

### 2.2. Hyperuricemia

The laboratory processes and methodologies for determining SUA levels were detailed on the NHANES website (https://www.cdc.gov/nchs/nhanes/). The NHANES database, which uses standardized laboratory procedures, provided the serum creatinine data used in this investigation. NHANES documentation states that the enzymatic method – more especially, the creatininase/sarcosine oxidase assay – was employed for measurements. The estimated glomerular filtration rate (eGFR) values in this NHANES-based study were derived using the 2009 Chronic Kidney Disease Epidemiology Collaboration creatinine equation, as standardized by NHANES for all cycles (2007–2018). HUA is defined as SUA levels above 416 µmol/L (7 mg/dL) in men and 357 µmol/L (6 mg/dL) in women, based on diagnostic criteria.^[18]^

### 2.3. Sitting time

In this study, the daily ST was measured using the following question as the exposure variable: “How much time do you usually spend sitting (or reclining) on a typical day?” Note that the duration of sleep is not included in this variable. Less than 4 hours per day, 4 to 6 hours per day, 6 to 8 hours per day, and 8 hours or more per day were the 4 categories into which the daily ST was typically classified.^[19,20]^

### 2.4. Covariates

To mitigate potential bias, several covariates were selected using prior findings from the pertinent literature,^[21–23]^ which including age, sex (male or female), race (Mexican American, other Hispanic, non-Hispanic White, non-Hispanic Black, and other/multiracial), educational level (<9th grade, 9–11th grade, high school graduate, some college, and college graduate or above), marital status (married/living with partner and widowed/divorced/separated/never married), poverty-to-income ratio (<1.29, 1.30–3.49, and ≥3.50), body mass index (BMI) (<25, ≥25, and <30, and ≥30 kg/m^2^), smoking and alcohol consumption status and the presence or absence of hypertension, diabetes, coronary heart disease (CHD) or cancer/malignancy. Furthermore, PA was collected as a confounding factor to be considered by participants, self-reported using the World Health Organization’s (WHO) Global Physical Activity Questionnaire, which was created by the WHO. According to WHO guidelines, each participant’s PA was converted to MET minutes of moderate to vigorous activity per week. The type of exercise affected the MET values, and NHANES gave reference MET values for every PA. PA score was computed based on weekly activity type, frequency, and duration using the formula: MET times weekly frequency multiplied by the duration of each PA.^[24]^

### 2.5. Statistical analysis

Sample weights were incorporated as advised by NHANES to ensure data representativeness, given the complexity of data collection in the NHANES database. Data analysis was performed using R software version 4.3.2 (R Project for Statistical Computing, Vienna, Austria). Tests were conducted using a 2-tailed significance criterion of *P* < .05. Analysis of variance was employed to compare group differences, presenting continuous variables as mean ± standard deviation to describe baseline characteristics. Frequencies and percentages were used to express categorical variables. The chi-square test was utilized to evaluate nominal categorical variables, and the Wilcoxon rank-sum test was employed to explore ordinal categorical variables.

Three models were employed in this study: model 1 had no modifications; model 2 was modified based on age, gender, race, educational background, marital status, and poverty-income ratio; and model 3 was modified from model 2, adding BMI, smoking and drinking status, history of hypertension, diabetes, CHD, and cancer/malignancy. Logistic regression analysis yielded odds ratios and corresponding 95% confidence intervals. The study employed restricted cubic spline (RCS) analysis to examine nonlinear relationships between HUA and daily ST, as well as PA. A stratified logistic regression analysis was conducted to examine interaction effects between covariates and daily ST, identifying variables that modify the relationship between ST and HUA across different PA subgroups. Finally, a mediation analysis was undertaken to evaluate the involvement of renal function-related indicators (serum creatinine, eGFR, and blood urea nitrogen) in the association between daily ST and HUA.

## 3. Results

### 3.1. Baseline characteristics of the study population

Initially, 59,842 individuals from the NHANES between 2007 and 2018 were extracted in the study. The final study included 25,470 patients aged ≥20 years with complete information from the United States, meeting the study’s inclusion and exclusion criteria. Figure 1 shows a full flow chart. The median age of the participants was 47.48 years (weighted), with 51.12% females and 48.88% males. Among the 25,470 participants, 11,138 (68.85%) were non-Hispanic White, 15,223 (63.86%) were married or lived with a partner, 17,642 (69.27%) lived in households with a family poverty-income ratio over 3.5, and 9911 (38.03%) had a BMI ≥ 30 kg/m^2^. The median PA for PA was 3852.36 MET-min/week (weighted), 16,553 (69.76%) had PA more than or equal to 400, and 8917 (30.24%) had PA <400. During interviews, over 75% of participants reported current alcohol consumption, and over 25% reported current smoking. The prevalence rates of comorbidities were as follows: 10.3% (n = 2470) for CHD (106), 3.48% for cancer, 11.93% (n = 3953) for hypertension and diabetes mellitus, and 32.1% (n = 9271) for hypertension. About 25% of respondents reported sitting for 4 to 6 hours daily, while 33.1% of the entire population sat for more than 8 hours. Additionally, sedentary individuals had higher odds of being married, having a higher household income-to-poverty ratio, higher education levels, higher BMI, not smoking, being less physically active, and having higher rates of diabetes, cancer, and hypertension. HUA was observed in 12.54% of all participants. The prevalence of HUA was 11.22%, 10.91%, 13.12%, and 14.2% among individuals who sat <4, 4 to 6, 6 to 8, and >8 hours per day, respectively. Sitting for an extended period of time increases the risk of developing high uric acid hematologic illness (*P* < .001). Table 1 displays the baseline characteristics of participants, classified by quartile of daily ST.

**Table 1:** Baseline characteristics of participants by categories of daily sitting time (weighted).

Characteristic	Overall (N = 25,470)	Sitting time (h)/d				
		<4 (N = 6982)	4–6 (N = 6074)	6–8 (N = 3989)	≥8 (N = 8425)	*P* value
No. (weighed)	108,895,888	25,636,914	25,943,220	17,492,150	39,823,604	
Sitting time (h)/d	6.25 ± 0.05	2.28 ± 0.02	4.44 ± 0.01	6.28 ± 0.01	9.96 ± 0.03	<.001
Age (yr)	47.48 ± 0.25	45.58 ± 0.28	47.98 ± 0.41	48.92 ± 0.45	47.75 ± 0.31	<.001
Age group (%)						<.001
20–39 yr	8486 (36.17)	2419 (39.37)	2015 (36)	1279 (35.08)	2773 (34.7)	
40–59 yr	8408 (37.53)	2464 (38.93)	1884 (35.08)	1191 (34.19)	2869 (39.69)	
60–80 yr	8576 (26.30)	2099 (21.7)	2175 (28.91)	1519 (30.73)	2783 (25.61)	
Gender (%)						.8789
Male	12,556 (48.88)	3412 (48.93)	3039 (48.31)	1994 (49.15)	4111 (49.1)	
Female	12,914 (51.12)	3570 (51.07)	3035 (51.69)	1995 (50.85)	4314 (50.9)	
Race (%)						<.001
Mexican American	3731 (8.13)	1654 (14.43)	872 (8.18)	455 (6.54)	750 (4.74)	
Other Hispanic	2547 (5.40)	963 (8.35)	623 (5.57)	331 (4.23)	630 (3.92)	
Non-Hispanic White	11,138 (68.85)	2436 (59.47)	2679 (68.82)	1927 (72.22)	4096 (73.43)	
Non-Hispanic Black	5155 (10.18)	1273 (10.5)	1244 (10.19)	800 (9.72)	1838 (10.18)	
Other/multiracial	2899 (7.43)	656 (7.25)	656 (7.25)	476 (7.29)	1111 (7.73)	
Educational level (%)						<.001
<9th grade	2350 (4.61)	1116 (8.73)	549 (4.57)	267 (3.24)	418 (2.6)	
9–11th grade	3466 (10.04)	1219 (14.08)	882 (10.84)	513 (9.69)	852 (7.06)	
High school graduate	5833 (22.90)	1738 (27.67)	1497 (25.13)	934 (24.24)	1664 (17.8)	
Some college	7738 (31.89)	1883 (30.76)	1870 (32.11)	1284 (33.1)	2701 (31.95)	
College graduate or above	6083 (30.56)	1026 (18.77)	1276 (27.36)	991 (29.73)	2790 (40.59)	
Marital status (%)						.0065
Married/living with partner	15,223 (63.86)	4353 (64.94)	3705 (65.64)	2307 (62.71)	4858 (62.5)	
Widowed/divorced/separated/never married	10,247 (36.14)	2629 (35.06)	2369 (34.36)	1682 (37.29)	3567 (37.5)	
Poverty-income ratio	3.02 ± 0.04	2.57 ± 0.04	2.95 ± 0.04	3.02 ± 0.05	3.37 ± 0.04	<.001
Poverty-income ratio group (%)						<.001
<1.29	8005 (21.11)	2714 (28.77)	1919 (21.37)	1220 (20.73)	2152 (16.18)	
1.30–3.49	9637 (35.50)	2817 (39.97)	2408 (37.74)	1492 (36.34)	2920 (30.79)	
≥3.50	7828 (43.39)	1451 (31.26)	1747 (40.89)	1277 (42.93)	3353 (53.03)	
BMI (kg/m^2^)	29.16 ± 0.08	28.3 ± 0.11	28.68 ± 0.13	29.39 ± 0.17	29.92 ± 0.13	<.001
BMI group (%)						
≤25	7181 (28.98)	2106 (32.58)	1732 (29.83)	1108 (28.51)	2235 (26.31)	
25–30	8378 (32.99)	2446 (34.34)	2104 (34.83)	1234 (30.93)	2594 (31.83)	
≥30	9911 (38.03)	2430 (33.07)	2238 (35.34)	1647 (40.56)	3596 (41.86)	
PA (MET-min/wk)	3852.36 ± 71.84	6466.11 ± 182.51	4683.61 ± 130.19	3229.9 ± 97.69	1901.61 ± 50.93	<.001
PA group (%)						<.001
<400	8917 (30.24)	2000 (22.81)	1798 (24.92)	1401 (29.08)	3718 (39.01)	
≥400	16,553 (69.76)	4982 (77.19)	4276 (75.08)	2588 (70.92)	4707 (60.99)	
Renal function-related indicators						
Serum creatinine (µmol/L)	78.18 ± 0.27	74.77 ± 0.36	77.08 ± 0.34	79.19 ± 0.51	80.64 ± 0.5	<.0001
eGFR (mL/min/1.73 m^2^)	116.38 ± 0.46	113.38 ± 0.51	114.46 ± 0.58	115.73 ± 0.82	119.85 ± 0.89	<.0001
Blood urea nitrogen (mmol/L)	4.89 ± 0.03	4.7 ± 0.04	4.9 ± 0.04	4.97 ± 0.04	4.96 ± 0.03	<.0001
Smoking status (%)						<.001
Current	5166 (19.43)	1464 (22.11)	1313 (21.47)	821 (19.3)	1568 (16.43)	
Former	6268 (25.14)	1518 (22.77)	1514 (24.65)	1052 (26.39)	2184 (26.43)	
Never	14,036 (55.44)	4000 (55.12)	3247 (53.88)	2116 (54.32)	4673 (57.15)	
Drinking status (%)						<.001
Current	18,331 (77.63)	4754 (74.21)	4430 (78.09)	2903 (78.06)	6244 (79.35)	
Former	3720 (12.20)	1071 (13.19)	888 (12.12)	590 (12.4)	1171 (11.54)	
Never	3419 (10.16)	1157 (12.59)	756 (9.78)	496 (9.54)	1010 (9.11)	
Hypertension (%)						<.001
Yes	9271 (32.10)	2142 (25.85)	2200 (31.97)	1580 (34.63)	3349 (35.1)	
No	16,199 (67.90)	4840 (74.15)	3874 (68.03)	2409 (65.37)	5076 (64.9)	
Diabetes (%)						<.001
Yes	3953 (11.93)	948 (9.69)	898 (10.74)	661 (13.44)	1446 (13.48)	
No	21,517 (88.07)	6034 (90.31)	5176 (89.26)	3328 (86.56)	6979 (86.52)	
Coronary heart disease (%)						<.001
Yes	2470 (10.30)	237 (3.17)	199 (4.02)	435 (4.15)	237 (3.17)	
No	23,000 (89.70)	5837 (96.83)	3790 (95.98)	7990 (95.85)	5837 (96.83)	
Cancer/malignancy (%)						<.001
Yes	1061 (3.48)	483 (7.5)	607 (10.84)	486 (12.07)	894 (10.98)	
No	24,409 (96.52)	6499 (92.5)	5467 (89.16)	3503 (87.93)	7531 (89.02)	
Hyperuricemia (%)						<.001
Yes	3439 (12.54)	785 (11.22)	767 (10.91)	590 (13.12)	1297 (14.2)	
No	22,031 (87.46)	6197 (88.78)	5307 (89.09)	3399 (86.88)	7128 (85.8)	

BMI = body mass index, eGFR = estimated glomerular filtration rate, MET = metabolic equivalent, PA = physical activity.

### 3.2. Association of daily ST with HUA

We used weighted logistic regression models and stratified logistic regression to investigate the relationship between daily sitting duration and HUA, with persons sitting for fewer than 4 hours per day serving as the reference group. In univariate logistic regression analysis, stratified by PA, the results showed that both in PA < 400 MET-min/week and PA ≥ 400 MET-min/week, the likelihood of HUA in the sedentary group was higher than that in the non-sedentary group (*P*-values were <.001 and .0035, respectively). After adjusting for confounding factors, multivariate analysis was conducted, showing a similar pattern in models 2 and 3. In model 3, participants who were sedentary for more than 8 hours per day had a higher risk of HUA – 36% (PA < 400 MET-min/week) and 22% (PA ≥ 400 MET-min/week), although the trend was not statistically significant. Interestingly, for PA < 400 MET-min/week, the risk of HUA was higher in the 4 to 6 hours sedentary time group for PA < 400 MET-min/week (all *P* < .05). However, for PA ≥ 400 MET-min/week, the difference was not significant (all *P* > .05) (Table 2).

**Table 2:** Univariate and multivariate analyses of logistic regression model stratified by physical activity (weighted).

Variable	Sitting time (h)/d					
	<4 (OR, 95% CI), *P*	4–6 (OR, 95% CI), *P*	6–8 (OR, 95% CI), *P*	≥8 (OR, 95% CI), *P*	*P* for trend	*P* for interaction
PA < 400 MET-min/wk						.0332
Model 1	Reference	1.44 (1.18, 1.76), <.001	1.57 (1.28, 1.93), <.001	1.69 (1.43, 2.01), <.001	<.001	
Model 2	Reference	1.30 (1.06, 1.60), .0124	1.34 (1.08, 1.67), .0073	1.50 (1.25, 1.80), <.001	<.001	
Model 3	Reference	1.24 (1.00, 1.52), .0456	1.27 (1.02, 1.58), .0355	1.36 (1.13, 1.64), .0011	.6463	
PA ≥ 400 MET-min/wk						
Model 1	Reference	1.04 (0.91, 1.18), .5782	1.29 (1.12, 1.49), <.001	1.31 (1.17, 1.47), <.001	.0035	
Model 2	Reference	0.98 (0.86, 1.12), .7865	1.23 (1.06, 1.42), .0054	1.30 (1.15, 1.48), <.001	<.001	
Model 3	Reference	0.96 (0.84, 1.10), .5395	1.18 (1.01, 1.36), .0323	1.22 (1.07, 1.39), .0023	.0927	

Model 1 was unadjusted.

Model 2 was adjusted for age, gender, race, education level, marital status, and poverty-income ratio.

Model 3 was adjusted for age, gender, race, education level, marital status, poverty-income ratio, BMI, smoking status, drinking status, hypertension, diabetes, coronary heart disease, and cancer/malignancy.

BMI = body mass index, CI = confidence interval, MET = metabolic equivalent, OR = odds ratio, PA = physical activity.

### 3.3. Nonlinear relationships

**Figure 2.**
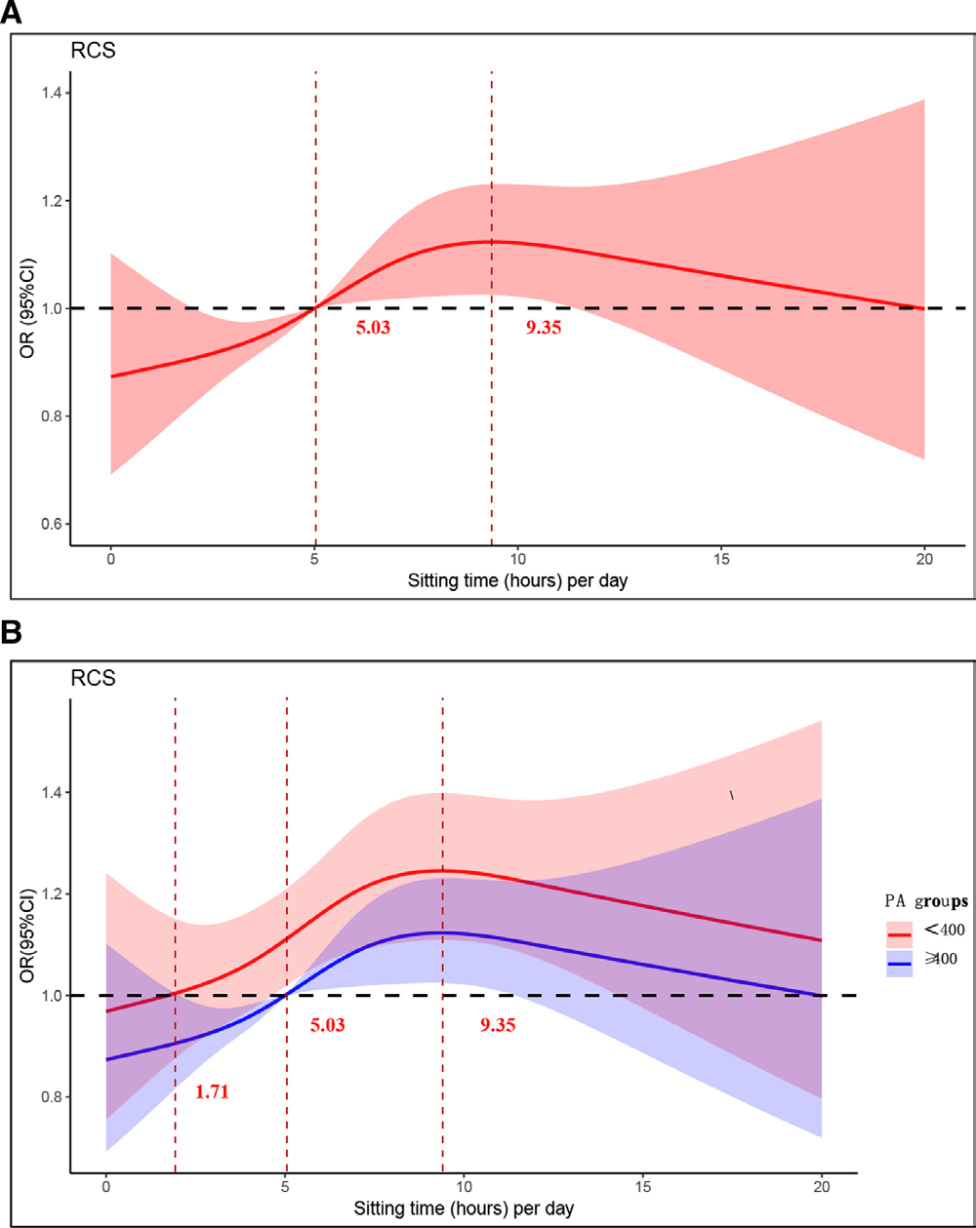
Fitting with restricted cubic spline for the relationship between daily sitting time and hyperuricemia. (A) Representing the overall situation, the 95% CI is shown by the transparent area, while the OR is indicated by the red line. (B) Represent the situation stratified by PA, the 95% CI is shown by the transparent area. The OR of <400 MET-min/week is indicated by the red line, while the OR of ≥400 MET-min/week is indicated by the blue line. Model 3 was used to alter these analyses. CI = confidence interval, MET = metabolic equivalent, OR = odds ratio, PA = physical activity, RCS = restricted cubic spline.

RCS models were employed to assess the dose-response connection between daily ST, PA levels, and HUA. In the fully adjusted model, daily ST showed a nonlinear dose-response relationship with HUA (*P* nonlinear <.05) and exhibited an inverted U-shaped pattern (Fig. 2). As daily ST increased, the risk of HUA gradually rose, peaking at 9.35 hours. Following prolonged daily sitting, the risk decreased gradually, yet remained positively correlated. According to the RCS curves depicting PA levels and HUA (Figure S1, Supplemental Digital Content, https://links.lww.com/MD/R229), the association between PA and HUA was nonlinear (*P* nonlinear <.05) and inversely correlated. As PA levels increased, the risk decreased gradually, reaching a nadir before slowly increasing, yet the association remained inverse.

### 3.4. Subgroup analyses

We utilized stratified logistic regression with interaction analysis to examine how PA influences the relationship between daily ST and HUA. In the PA < 400 MET-min/week group, we observed an interaction between hypertension and daily ST, with the highest risk of HUA observed in the 4 to 6-hour group, although the trend was not specific. Furthermore, the results showed that males, <9th grade, a poverty-to-income ratio of <1.29, BMI ≥ 30 kg/m^2^, current smokers, occasional and nondrinkers, with hypertension, diabetes, and cancer, had a higher likelihood of HUA in the PA < 400 MET-min/week group (Table 3).

**Table 3:** Stratified analyses in PA < 400 MET-min/week.

Characteristic	Sitting time (h)/d					
	<4 (OR, 95% CI), *P*	4–6 (OR, 95% CI), *P*	6–8 (OR, 95% CI), *P*	≥8 (OR, 95% CI), *P*	*P* for trend	*P* for interaction
Age group (%)						.3014
20–39 yr	Reference	0.84 (0.46, 1.53), .5654	1.00 (0.54, 1.86), .9909	0.93 (0.55, 1.56), .7848	.9368	
40–59 yr	Reference	1.45 (0.93, 2.25), .0996	1.86 (1.20, 2.89), .0056	1.51 (1.02, 2.22), .0373	.0634	
60–80 yr	Reference	1.16 (0.89, 1.52), .2694	1.00 (0.75, 1.34), .9969	1.13 (0.89, 1.45), .3131	.5159	
Gender (%)						.9855
Male	Reference	1.20 (0.91, 1.58), .2039	1.16 (0.87, 1.55), .3027	1.19 (0.93, 1.52), .1590	.2625	
Female	Reference	1.13 (0.81, 1.57), .4742	1.07 (0.75, 1.53), .7054	1.12 (0.83, 1.52), .4603	.5810	
Race (%)						.7325
Mexican American	Reference	1.39 (0.81, 2.39), .2300	1.97 (1.13, 3.43), .0169	1.23 (0.71, 2.11), .4625	.2422	
Other Hispanic	Reference	1.25 (0.64, 2.44), .5139	1.31 (0.64, 2.68), .4592	1.40 (0.78, 2.54), .2628	.2784	
Non-Hispanic White	Reference	0.91 (0.64, 1.30), .6119	0.90 (0.63, 1.29), .5657	1.00 (0.73, 1.36), .9849	.7319	
Non-Hispanic Black	Reference	1.50 (1.00, 2.25), .0480	1.16 (0.74, 1.82), .5169	1.29 (0.89, 1.86), .1732	.5078	
Other/multiracial	Reference	1.10 (0.55, 2.21), .7947	1.12 (0.53, 2.34), .7668	1.11 (0.59, 2.07), .7517	.7874	
Educational level (%)						.2304
<9th grade	Reference	1.05 (0.63, 1.75), .8572	1.70 (1.01, 2.85), .0448	1.26 (0.78, 2.03), .3430	.1879	
9–11th grade	Reference	1.75 (1.09, 2.80), .0197	1.54 (0.93, 2.55), .0916	1.19 (0.76, 1.86), .4494	.8836	
High school graduate	Reference	0.95 (0.63, 1.44), .8157	1.10 (0.72, 1.69), .6687	1.14 (0.80, 1.64), .4678	.3131	
Some college	Reference	1.25 (0.80, 1.95), .3307	1.06 (0.66, 1.69), .8116	1.18 (0.80, 1.76), .4036	.6193	
College graduate or above	Reference	1.06 (0.57, 1.97), .8638	0.58 (0.29, 1.14), .1145	0.95 (0.56, 1.61), .8413	.8519	
Marital status (%)						.8171
Married/living with partner	Reference	1.21 (0.92, 1.60), .1746	1.18 (0.88, 1.58), .2754	1.25 (0.98, 1.60), .0762	.1258	
Widowed/divorced/separated/never married	Reference	1.18 (0.85, 1.64), .3307	1.14 (0.81, 1.61), .4388	1.08 (0.81, 1.45), .6039	.8718	
Poverty-income ratio group (%)						.9289
<1.29	Reference	1.08 (0.78, 1.49), .6477	1.03 (0.73, 1.47), .8539	1.06 (0.79, 1.42), .7022	.7871	
1.30–3.49	Reference	1.28 (0.92, 1.78), .1473	1.33 (0.94, 1.89), .1091	1.23 (0.91, 1.66), .1873	.3364	
≥3.50	Reference	1.38 (0.80, 2.37), .2475	1.25 (0.73, 2.16), .4184	1.39 (0.86, 2.24), .1760	.2868	
BMI group (%)						.3000
≤25	Reference	0.69 (0.37, 1.26), .2262	1.03 (0.57, 1.87), .9222	1.02 (0.61, 1.71), .9482	.4808	
25–30	Reference	1.11 (0.77, 1.58), .5795	1.04 (0.71, 1.53), .8391	1.03 (0.74, 1.43), .8564	1.000	
≥30	Reference	1.49 (1.11, 2.00), .0081	1.37 (1.01, 1.86), .0439	1.52 (1.17, 1.97), .0015	.0096	
Smoking status (%)						.4759
Current	Reference	1.78 (1.07, 2.97), .0263	1.22 (0.70, 2.12), .4858	1.43 (0.89, 2.29), .1378	.1744	
Former	Reference	0.96 (0.66, 1.40), .8400	1.20 (0.82, 1.77), .3410	1.13 (0.81, 1.57), .4866	.9892	
Never	Reference	1.17 (0.87, 1.58), .3027	1.13 (0.82, 1.55), .4546	1.14 (0.87, 1.48), .3505	.6798	
Drinking status (%)						.4497
Current	Reference	1.01 (0.78, 1.31), .9458	1.10 (0.84, 1.44), .4979	1.14 (0.91, 1.44), .2637	.0500	
Former	Reference	1.62 (0.98, 2.66), .0578	1.17 (0.69, 2.00), .5625	1.19 (0.75, 1.89), .4547	.3106	
Never	Reference	1.55 (0.93, 2.59), .0945	1.33 (0.75, 2.35), .3229	1.22 (0.76, 1.97), .4116	.4907	
Hypertension (%)						.0489
Yes	Reference	1.36 (1.04, 1.76), .0233	1.13 (0.85, 1.49), .3992	1.17 (0.92, 1.48), .2094	.7369	
No	Reference	0.87 (0.60, 1.25), .4465	1.24 (0.86, 1.79), .2560	1.22 (0.89, 1.66), .2105	.0739	
Diabetes (%)						.0828
Yes	Reference	1.83 (1.20, 2.79), .0047	1.48 (0.94, 2.33), .0905	1.73 (1.18, 2.54), .0054	.0499	
No	Reference	1.00 (0.78, 1.29), .9770	1.07 (0.83, 1.38), .6131	1.03 (0.83, 1.28), .8036	.7554	
Coronary heart disease (%)						.2816
Yes	Reference	0.89 (0.50, 1.58), .6819	0.65 (0.36, 1.19), .1625	0.84 (0.49, 1.42), .5096	.5523	
No	Reference	1.21 (0.97, 1.52), .0960	1.24 (0.97, 1.58), .0797	1.21 (0.99, 1.49), .0601	.1204	
Cancer/malignancy (%)						.5025
Yes	Reference	1.44 (0.66, 3.14), .3638	0.86 (0.39, 1.91), .7102	1.15 (0.58, 2.27), .6926	.9883	
No	Reference	1.16 (0.93, 1.44), .1967	1.20 (0.95, 1.52), .1177	1.18 (0.97, 1.44), .0979	.1479	

Model 3 was adjusted for age, gender, race, education level, marital status, poverty-income ratio, BMI, smoking status, drinking status, hypertension, diabetes, coronary heart disease, and cancer/malignancy. All models were adjusted for all variables other than themselves.

BMI = body mass index, CI = confidence interval, MET = metabolic equivalent, OR = odds ratio, PA = physical activity.

### 3.5. Mediation analyses

**Figure 3.**
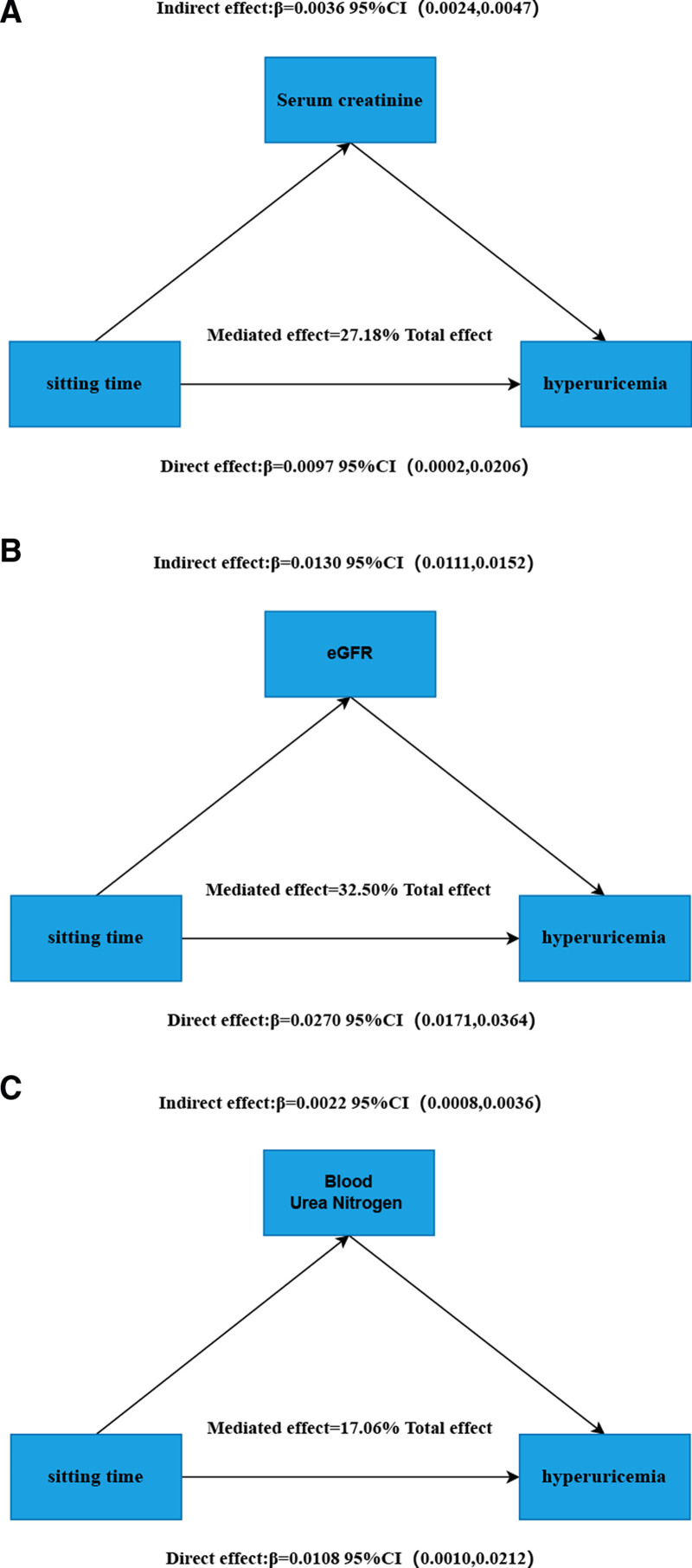
Estimated mediated proportions of associations between daily sitting time and hyperuricemia. Model 3 was used for these analyses. (A) Serum creatinine–mediated proportion (27.18%). (B) eGFR-mediated proportion (32.50%). (C) Blood urea nitrogen–mediated proportion (17.06%). CI = confidence interval, eGFR = estimated glomerular filtration rate.

This study identified strong associations between higher serum creatinine, elevated blood urea nitrogen, and lower eGFR with an increased risk of HUA (Tables S1, S3, and S5, Supplemental Digital Content, https://links.lww.com/MD/R229). Additionally, prolonged sitting duration was significantly associated with elevated serum creatinine, blood urea nitrogen, and poorer eGFR (Tables S2, S4, and S6, Supplemental Digital Content, https://links.lww.com/MD/R229). Consequently, we conducted a mediation analysis to examine the role of renal function-related variables (serum creatinine, eGFR, and blood urea nitrogen) in the association between daily sitting duration and HUA (Table 4). According to the findings, 27.18% of the relationship between daily sitting duration and HUA was mediated by serum creatinine, 32.50% of the relationship between daily ST and HUA was mediated by eGFR, 17.06% of the relationship between daily sitting duration and HUA was mediated by blood urea nitrogen, and each shown an insufficient mediating effect (*P* mediation <.05) (Fig. 3).

**Table 4:** Mediation effects of renal function-related indicators on the association of sitting time and hyperuricemia.

Exposure	Model 1			Model 2			Model 3		
	β (95% CI)	*P* value		β (95% CI)	*P* value		β (95% CI)		*P* value
Serum creatinine (µmol/L)									
Direct effect	0.0327 (0.0224, 0.0437)	<.0001		0.0332 (0.0236, 0.0441)	<.0001		0.0097 (0.0002, 0.0206)		.005
Indirect effect	0.0070 (0.0036, 0.0119)	<.0001		0.0045 (0.0029, 0.0058)	<.0001		0.0036 (0.0024, 0.0047)		<.0001
Total effect	0.0397 (0.0293, 0.0500)	<.0001		0.0377 (0.0277, 0.0484)	<.0001		0.0133 (0.0033, 0.0241)		.01
Percent mediation (%) (95% CI)	17.59 (9.03, 30.57)			11.88 (7.41, 17.16)			27.18 (13.01, 92.22)		
*P* value	<.0001			<.0001			.01		
eGFR (mL/min/1.73 m^2^)									
Direct effect	0.0270 (0.0171, 0.0364)		<.0001	0.0251 (0.0151, 0.0347)		<.0001	0.0270 (0.0171, 0.0364)	<.0001	
Indirect effect	0.0130 (0.0111, 0.0152)		<.0001	0.0116 (0.0094, 0.0139)		<.0001	0.0130 (0.0111, 0.0152)	<.0001	
Total effect	0.0400 (0.0302, 0.0496)		<.0001	0.0367 (0.0260, 0.0465)		<.0001	0.0400 (0.0302, 0.0496)	<.0001	
Percent mediation (%) (95% CI)	32.50 (24.87, 44.06)			31.58 (23.62, 44.10)			32.50 (24.87, 44.06)		
*P* value	<.0001			<.0001			<.0001		
Blood urea nitrogen (mmol/L)									
Direct effect	0.0328 (0.0228, 0.0431)		<.0001	0.0332 (0.0231, 0.0443)		<.0001	0.0108 (0.001, 0.0212)	.034	
Indirect effect	0.0073 (0.0054, 0.0091)		<.0001	0.0043 (0.0027, 0.0058)		<.0001	0.0022 (0.0008, 0.0036)	<.0001	
Total effect	0.0401 (0.0300, 0.0507)		<.0001	0.0375 (0.0274, 0.0484)		<.0001	0.0130 (0.0032, 0.0238)	.012	
Percent mediation (%) (95% CI)	18.21 (12.81, 25.50)			11.37 (6.91, 17.34)			17.06 (4.89, 62.03)		
*P* value	<.0001			<.0001			.012		

Model 1 was unadjusted.

Model 2 was adjusted for age, gender, race, education level, marital status, and poverty-income ratio.

Model 3 was adjusted for age, gender, race, education level, marital status, poverty-income ratio, BMI, smoking status, drinking status, hypertension, diabetes, coronary heart disease, and cancer/malignancy.

BMI = body mass index, CI = confidence interval, eGFR = estimated glomerular filtration rate.

## 4. Discussion

The analysis of 25,470 individuals (NHANES 2007–2018) revealed significant associations between SB and HUA. Participants with prolonged ST (≥8 h/day) exhibited higher HUA prevalence than ST (<4 h/day) (14.2% vs 11.22%; *P* < .001). Weighted logistic regression demonstrated an inverted U-shaped dose-response relationship (*P* nonlinear <.05), with peak HUA risk at 9.35 daily sitting hours. PA significantly modified this association: individuals with PA ≥ 400 MET-min/week showed attenuated risk (22% elevated risk for ≥8 h/day), whereas those with PA < 400 MET-min/week had substantially higher odds (36% risk elevation, *P* < .05), particularly in the 4 to 6 h/day subgroup. Stratified analyses identified high-risk profiles among less active adults with hypertension, obesity (BMI ≥ 30 kg/m^2^), or lower socioeconomic status. Critically, renal biomarkers mediated the sitting-HUA relationship: serum creatinine explained 27.18% of the association, eGFR mediated 32.50%, and blood urea nitrogen accounted for 17.06% (all *P* mediation <.05). These findings underscore SB as an independent risk factor for HUA, partially operating through renal dysfunction pathways.

A significant portion of the adult population participates in sedentary activities, thereby increasing the risk of various chronic diseases. According to a systematic review, SB is prevalent in both high-income and low-income nations, influenced by factors such as age, gender, socioeconomic status, and occupation.^[25]^ Du et al analyzed trends in SB among US adults from 2007 to 2016, revealing an increase in self-reported ST across all demographic categories, underscoring the escalating public health concern associated with sedentary lifestyles.^[26]^ There is a paucity of studies on the association between SB and HUA in the US population. Therefore, this study aimed to explore the association between ST and HUA in US adults and employ mediation analysis. Overall, our results showed a nonlinear relationship between ST and HUA, with an increasing trend in the adjusted prevalence of HUA as ST increased (*P* < .05). This finding aligns with those of the Chinese multi-ethnic cohort study.^[27]^ Prolonged sitting can slow the body’s metabolism, affecting purine metabolism speed and potentially increasing uric acid production, thus disrupting purine balance in the body. Purine serves as a uric acid precursor, and metabolic dysregulation can elevate uric acid levels.^[27,28]^

Moreover, inactivity associated with chronic inflammation and inflammatory reactions may influence uric acid levels in the body, leading to elevated blood uric acid.^[29]^ A study in South Korea found that people who spent ≥10 h/day in SB were more likely to have HUA than those who spent <5 h/day.^[29]^ Our study’s RCS also yielded similar findings, revealing that after adjusting for confounding variables, HUA steadily decreased but remained significantly associated at 9.35 hours of daily sitting. The saturation point of sedentary time in the RCS results of model 3 for PA still applies, whether in the group with PA ≥400 MET-min/week or <400 MET-min/week. The hierarchical logistic regression analysis of PA and the RCS results indicates an interaction between PA and ST in the relationship with HUA. PA appears to mitigate the elevated risk of HUA associated with prolonged sitting, particularly in the 4 to 6-hour group. This result was validated in further stratified logistic regression analysis of PA. Additionally, a Chinese cohort study reported an association between PA and SB, noting that the protective effect of PA on HUA became nonsignificant when daily ST exceeded 6 hours.^[30]^ A similar pattern was observed regarding the protective impact of PA against increased mortality associated with prolonged sitting. Elevated levels of moderate-intensity PA appear to mitigate the higher risk of death linked to prolonged sitting, according to a harmonized meta-analysis of data from over a million men and women.^[31]^ A cross-sectional study showed that moderate PA was beneficial in mitigating subclinical atherosclerosis among individuals with low levels of daily sitting (<3 h/day). However, no significant health benefits were observed when daily ST exceeded 3 h/day.^[32]^

Furthermore, our stratified analysis revealed no statistically significant differences in the association between daily ST and HUA based on gender or BMI. This differs from the results of other researchers. Previous research indicates that moderate PA may offer greater benefits in reducing the risk of elevated blood uric acid levels, suggesting that reducing sedentary time could be more impactful for preventing HUA in women compared to men.^[27]^ Several factors could explain this disparity. Interestingly, in our stratified analysis, never-drinkers and occasional drinkers were more likely to develop HUA. Limitations due to our inability to correct for dietary factors. This may stem from compensatory high-fructose beverage consumption and loss of renal vasodilatory effects from moderate alcohol intake. Crucially, this subgroup exhibited higher rates of obesity and hypertension – synergistically impairing urate excretion via insulin resistance and reduced renal perfusion. Future studies should quantify dietary purine/fructose intake to clarify this association. First, the populations in our study and previous studies differed, potentially contributing to racial disparities. Second, this study conducted a stratified analysis based on PA stratification, which differed from the stratified analysis of all participants. Therefore, more comprehensive ethnic studies are necessary to explore these differences.

Previous research has found that prolonged sitting is connected with an increased risk of a variety of renal disorders, including proteinuria, CKD, dialysis, and mortality from all causes, CHD, and kidney disease.^[33,34]^ The results of linear regression in our study also revealed that SB was connected with the increase of serum creatinine and SUA, as well as the decrease of eGFR. Although whether HUA is a prediction of CKD or just a consequence of diminished uric acid excretion is still unclear, this subject is still a source of contention.^[35,36]^ However, there’s little question that HUA can result from renal injury.^[37]^ We hypothesized that the relationship between daily sitting duration and HUA may be mediated by associated renal function measures. This study demonstrated that longer ST, higher serum creatinine, higher blood urea nitrogen, and lower eGFR values were strongly related to a greater incidence of HUA. Furthermore, the results indicated that serum creatinine, blood urea nitrogen, and eGFR all partially mediated the connection between the daily ST and HUA. Some probable explanations for the intricate relationships: sedentism reduces total blood volume and blood flow circulation, and when endothelium-dependent blood channel relaxation capacity declines, vascular endothelial damage from blood flow resistance increases.^[38,39]^ As a result, continuing SB may raise the risk of CKD due to abnormal blood flow circulation in the kidney or vascular structure.^[39,40]^ Individuals with renal disease often have elevated uric acid levels.^[37,41]^

This study’s strength is the use of a large, nationwide US population cohort that was followed over time. Furthermore, we employed comprehensive RCS and subgroup analyses to visually demonstrate the association between ST and HUA, thereby enhancing the reliability of our study results. Additionally, to the best of our knowledge, this is the 1st study to investigate the relationship between daily sitting duration and HUA, including interaction and mediation analyses. However, there are several shortcomings with our study. The cross-sectional study design of our research, which is based on the NHANES database, makes it challenging to identify the cause of the link. Additionally, our adjustment may not have been comprehensive enough, as we did not include data on certain risk factors for HUA, such as dietary habits and daily water intake, as covariates. In the analysis of PA, we calculated the relationship between total PA and daily ST, and did not classify PA to conduct the interaction analysis, which may contribute to bias. With regard to exclusion criteria, we cannot currently exclude certain diseases and medications that could directly affect SUA and PA based on NHANES data. Therefore, additional studies are needed to confirm our findings and investigate the underlying mechanisms.

## 5. Conclusion

In conclusion, this study among the US adult population revealed a rising prevalence of HUA associated with increased daily ST, particularly evident among those with <400 MET-min/week of PA. We observed an inverted U-shaped relationship between daily sitting duration and HUA. Furthermore, serum creatinine, blood urea nitrogen, and eGFR may play crucial roles as mediators linking daily sitting duration to increased HUA. Further research is necessary to deepen our understanding of the intricate relationship between SB and HUA, including exploring the underlying mechanisms.

## Acknowledgments

The authors are grateful to the NHANES workers and participants for their excellent contributions.

## Author contributions

**Methodology:** Li Li, Fei Wang.

**Data curation:** Fei Wang.

**Investigation:** Fei Wang.

**Supervision:** Qifang Guo.

**Visualization:** Qifang Guo.

**Writing – original draft:** Li Li.

**Writing – review & editing:** Qifang Guo.

## Supplementary Material


