# Supplementary material for: Daily sitting time and hyperuricemia in US adults: A dose-response association with the mediation effect of renal function-related indicators (NHANES 2007–2018)
Source: Medicine (Baltimore). 2026 Jan 30;105(5):e46026. doi: 10.1097/MD.0000000000046026 (PMC12863912; doi:10.1097/MD.0000000000046026)
Supplement: Supplementary file 1 [file medi-105-e46026-s001.docx]

**Table S1**  **The relationship between** **Serum creatinine and hyperuricemia among participants from the NHANES (2007-2018).**

| **Characteristic** | **Model 1** | |  | **Model 2** | |  | **Model 3** | |
| --- | --- | --- | --- | --- | --- | --- | --- | --- |
|  | **OR (95% CI)** | **P value** |  | **OR (95% CI)** | **P value** |  | **OR (95% CI)** | **P value** |
| **Serum creatinine(umo/L)** | 1.03(1.02,1.03) | <0.0001 |  | 1.02(1.01,1.02) | <0.0001 |  | 1.01（1.01,1.02） | <0.0001 |

Model 1 was unadjusted.

Model 2 was adjusted for age, gender, race, education level, marital status, and poverty-income ratio.

Model 3 was adjusted for age, gender, race, education level, marital status, poverty-income ratio, BMI, smoking status, drinking status, hypertension, diabetes, coronary heart disease and cancer/malignancy.

Abbreviations: OR, odds ratio; CI, Confidence interval.

**Table S2 The relationship between Serum creatinine and Sitting time among participants from the NHANES (2007-2018).**

| **Characteristic** | **Model 1** | |  | **Model 2** | |  | **Model 3** | |
| --- | --- | --- | --- | --- | --- | --- | --- | --- |
|  | **β (95% CI)** | **P value** |  | **β (95% CI)** | **P value** |  | **β (95% CI)** | **P value** |
| **Sitting time（hours）/day** | 0.65（0.52,0.79） | <0.0001 |  | 0.51（0.38,0.64） | <0.0001 |  | 0.46（0.34,0.58） | <0.0001 |

Model 1 was unadjusted.

Model 2 was adjusted for age, gender, race, education level, marital status, and poverty-income ratio.

Model 3 was adjusted for age, gender, race, education level, marital status, poverty-income ratio, BMI, smoking status, drinking status, hypertension, diabetes, coronary heart disease and cancer/malignancy.

Abbreviations: CI, Confidence interval.

**Table S3 The relationship between eGFR and hyperuricemia among participants from the NHANES (2007-2018).**

| **Characteristic** | **Model 1** | |  | **Model 2** | |  | **Model 3** | |
| --- | --- | --- | --- | --- | --- | --- | --- | --- |
|  | **OR (95% CI)** | **P value** |  | **OR (95% CI)** | **P value** |  | **OR (95% CI)** | **P value** |
| **eGFR** **(mL/min/1.73 m ^2^ )** | 0.97（0.97,0.98） | <0.0001 |  | 0.96（0.96,0.97） | <0.0001 |  | 0.96（0.96,0.97） | <0.0001 |

Model 1 was unadjusted.

Model 2 was adjusted for age, gender, race, education level, marital status, and poverty-income ratio.

Model 3 was adjusted for age, gender, race, education level, marital status, poverty-income ratio, BMI, smoking status, drinking status, hypertension, diabetes, coronary heart disease and cancer/malignancy.

Abbreviations: eGFR, estimated glomerular filtration rate; OR, odds ratio; CI, Confidence interval.

**Table S4 The relationship between eGFR and Sitting time among participants from the NHANES (2007-2018).**

| **Characteristic** | **Model 1** | |  | **Model 2** | |  | **Model 3** | |
| --- | --- | --- | --- | --- | --- | --- | --- | --- |
|  | **β (95% CI)** | **P value** |  | **β (95% CI)** | **P value** |  | **β (95% CI)** | **P value** |
| **Sitting time（hours）/day** | -0.53（-0.65,-0.41） | <0.0001 |  | -0.29（-0.31,-0.21） | <0.0001 |  | -0.26（-0.34,-0.19） | <0.0001 |

Model 1 was unadjusted.

Model 2 was adjusted for age, gender, race, education level, marital status, and poverty-income ratio.

Model 3 was adjusted for age, gender, race, education level, marital status, poverty-income ratio, BMI, smoking status, drinking status, hypertension, diabetes, coronary heart disease and cancer/malignancy.

Abbreviations: eGFR, estimated glomerular filtration rate; CI, Confidence interval.

**Table S5 The relationship between Blood Urea Nitrogen and hyperuricemia among participants from the NHANES (2007-2018).**

| **Characteristic** | **Model 1** | |  | **Model 2** | |  | **Model 3** | |
| --- | --- | --- | --- | --- | --- | --- | --- | --- |
|  | **OR (95% CI)** | **P value** |  | **OR (95% CI)** | **P value** |  | **OR (95% CI)** | **P value** |
| **Blood Urea Nitrogen(mmol/L)** | 1.28（1.25,1.30） | <0.0001 |  | 1.27（1.24,1.30） | <0.0001 |  | 1.27（1.24,1.30） | <0.0001 |

Model 1 was unadjusted.

Model 2 was adjusted for age, gender, race, education level, marital status, and poverty-income ratio.

Model 3 was adjusted for age, gender, race, education level, marital status, poverty-income ratio, BMI, smoking status, drinking status, hypertension, diabetes, coronary heart disease and cancer/malignancy.

Abbreviations: OR, odds ratio; CI, Confidence interval.

**Table S6 The relationship between Blood Urea Nitrogen and Sitting time among participants from the NHANES (2007-2018).**

| **Characteristic** | **Model 1** | |  | **Model 2** | |  | **Model 3** | |
| --- | --- | --- | --- | --- | --- | --- | --- | --- |
|  | **β (95% CI)** | **P value** |  | **β (95% CI)** | **P value** |  | **β (95% CI)** | **P value** |
| **Sitting time（hours）/day** | 0.01（0.02,0.04） | <0.0001 |  | 0.01（0.00,0.02） | 0.0053 |  | 0.01（0.00,0.02） | 0.1605 |

Model 1 was unadjusted.

Model 2 was adjusted for age, gender, race, education level, marital status, and poverty-income ratio.

Model 3 was adjusted for age, gender, race, education level, marital status, poverty-income ratio, BMI, smoking status, drinking status, hypertension, diabetes, coronary heart disease and cancer/malignancy.

Abbreviations: CI, Confidence interval.

**Figure S1 Fitting with restricted cubic spline for the relationship between physical activity and hyperuricemia.**


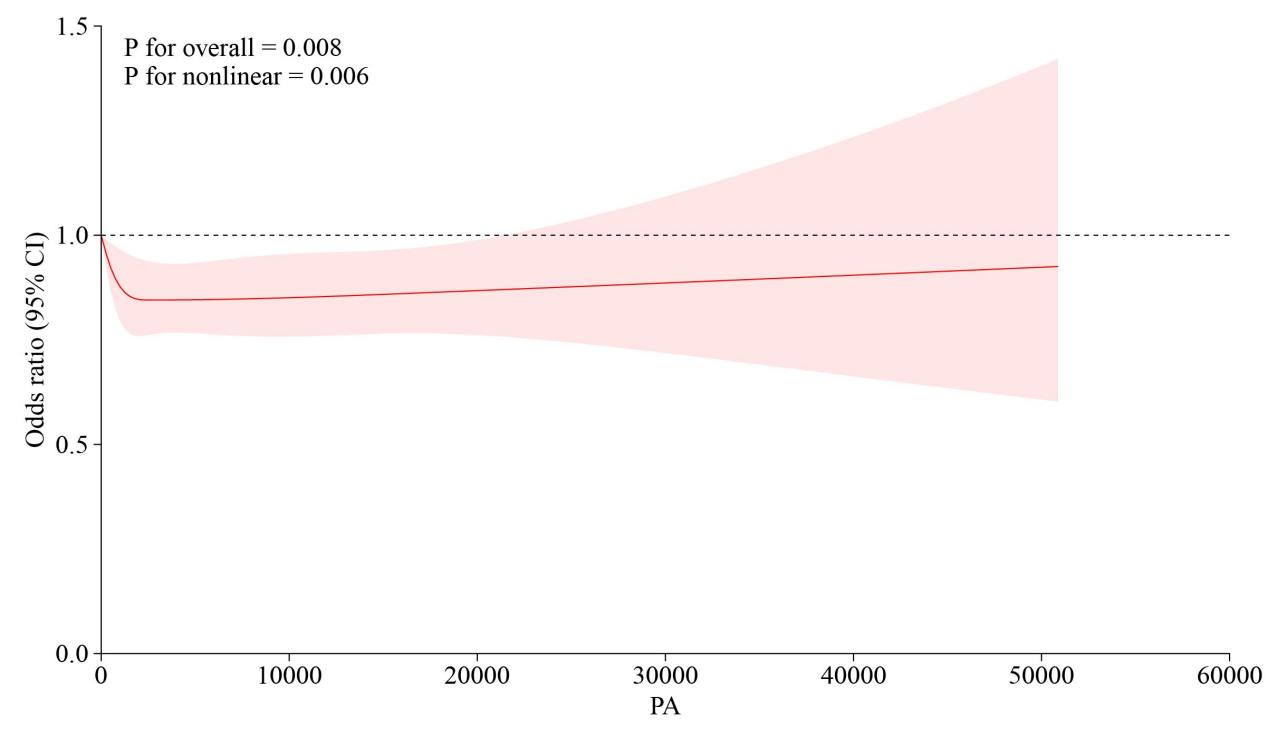


The 95% CI is shown by the transparent area, while the OR is indicated by the red line. Model 3 was used to alter these analyses. Abbreviations: PA, Physical activity; OR, Odds ratio.
